# Supplementary material for: The First K+-Channel Blocker Described from Tityus fasciolatus Venom: The Purification, Molecular Cloning, and Functional Characterization of α-KTx4.9 (Tf5)
Source: Toxins (Basel). 2025 Feb 18;17(2):96. doi: 10.3390/toxins17020096 (PMC11861696; doi:10.3390/toxins17020096)
Supplement: Supplementary file 1 [file toxins-17-00096-s001.zip › toxins-3470559-supplementary.pdf]

The first K+-channel blocker described from *Tityus fasciolatus* venom: Purification, molecular cloning and functional characterization of α-KTx4.9 (Tf5)

Suplementar data

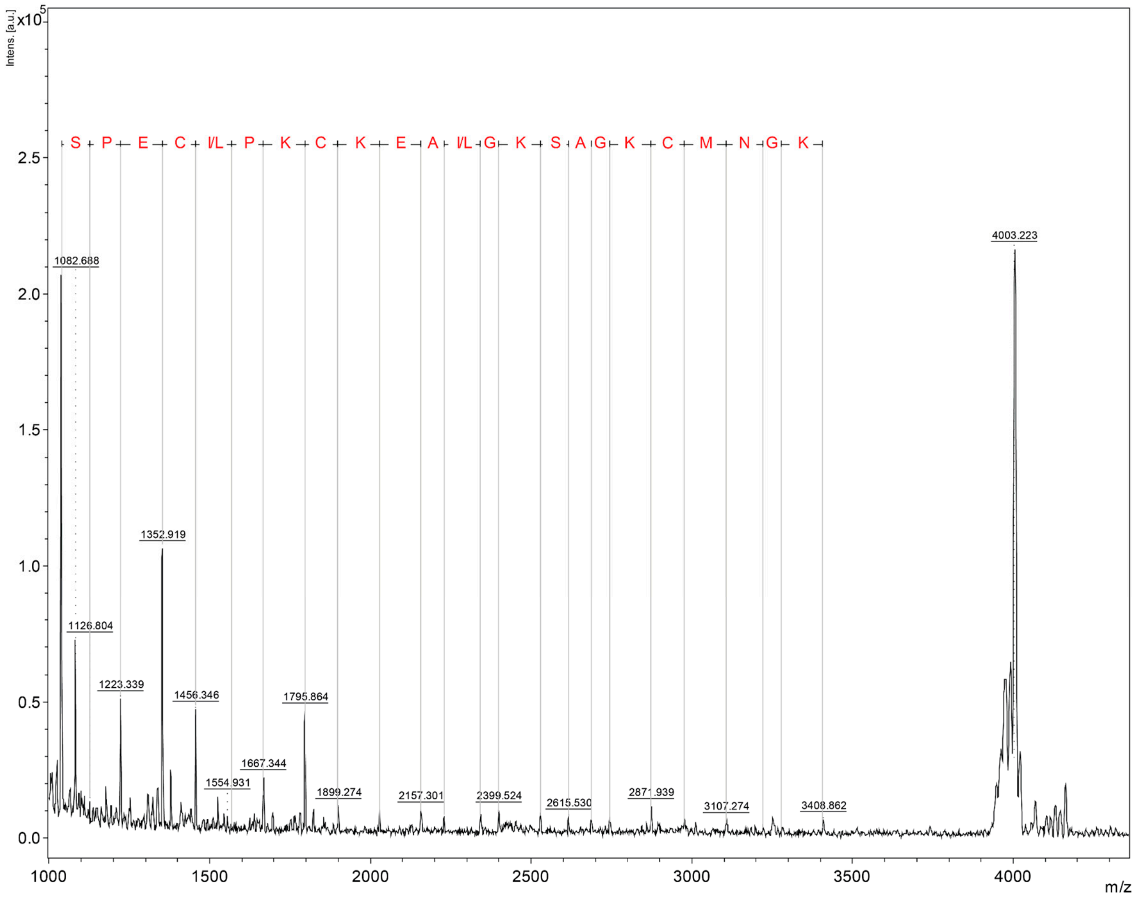

Figure S1 - Sequence fragment of Tf5 toxin. Sequence obtained by In source decay mass spectrometry method
